# Editorial: *In vivo* and *in vitro* control of infectious parasitic diseases

**DOI:** 10.3389/fmicb.2022.1062904

**Published:** 2022-10-19

**Authors:** Mohamed A. Dkhil, Rewaida Abdel-Gaber, Xiaoping Chen

**Affiliations:** ^1^Department of Zoology and Entomology, Faculty of Science, Helwan University, Cairo, Egypt; ^2^Department of Zoology, Faculty of Science, Cairo University, Giza, Egypt; ^3^Guangzhou Institutes of Biomedicine and Health, Chinese Academy of Sciences (CAS), Guangzhou, China

**Keywords:** parasitic diseases, vaccination, natural products, nanoparticles, host-parasite relationship

Infection with parasites results in significant fatalities and significant financial losses. Particularly in underdeveloped nations in Africa and Southeast Asia, parasitic infections cause a significant level of morbidity, mortality, and socioeconomic underdevelopment. The problem will be solved if we can find a way to control such infectious diseases.

Surra is a parasitic disease caused by the eukaryotic, unicellular hemoprotozoan, *Trypanosoma evansi*, which affects the development of animal production and is widespread among both domestic and wild animals. Therefore, Dkhil et al. focused on the finding of an alternative medicinal plant of *Eucalyptus camaldulensis* leaf extracts (ELE) for the treatment of *T. evani*-induced brain injury and spleen immune response in mice. ELE decreased the number of trypanosomes in the blood and improved the weight loss caused by infection. In addition, ELE reduced the parasite-induced brain and spleen histopathological damage. The parasite affected the levels of dopamine and serotonin, but after treatment with ELE, their concentrations significantly decreased to 154 ± 7 and 258 ± 11 μg/g, respectively. This study demonstrated the antiparasitic and ameliorative activity of ELE in the infected mice with *T. evani*.

Malaria infections are persistent as a frequent recrudescence of the disease may occur following the acute infection stage, but the different immune responses that control the acute and recrudescence stages are still largely unknown. Chen et al. used single-cell RNA sequencing (scRNA-seq) to follow the number of Th1 and plasma cells in the spleen that reduced during the recurrence stage compared to the acute stage of *Plasmodium chabaudi* AS (*P. chabaudi*) infection. In contrast, the number of innate immune cells, including red pulp macrophages (RPMs), gamma delta (γδ) T cells, and Dendritic cells (DCs) were significantly increased during the recurrence stage and showed to be critical for *P. chabaudi* infection recurrence control. This study suggests the complementary role of innate immune responses in controlling malaria recrudescence when adaptive immune responses are suppressed.

The global distribution of breast cancer and the opportunistic nature of the parasite has resulted in many patients with breast cancer can be infected with toxoplasmosis. Barakat et al. studied the potential effects of tamoxifen on chronic toxoplasmosis in animal models (Swiss albino mice). Tamoxifen increased the parasite burden on animals treated with the drug during 14 and 28 days as compared with the control group. Furthermore, treatment with tamoxifen-induced a series of histopathological and immunohistochemical changes in the kidney, liver, brain, and uterus, revealing the exacerbating effect of tamoxifen against chronic toxoplasmosis. This study demonstrated the potential role of tamoxifen during chronic toxoplasmosis, particularly in immunocompromised patients which could be life-threatening.

Schistosomes transmit from a freshwater habitat with limited resources to a mammalian host environment seeking a limitless supply of glucose. The regulatory mechanisms that control schistosome energy metabolism are poorly understood. Therefore, Hunter et al. focused on the finding of the function of AMPK in schistosomes and its role in the regulation of parasite glycolysis. When AMPK α was chemically inhibited, adult schistosomes mounted a compensatory response that enhanced AMPK α protein abundance and activity. Larval schistosomula are susceptible to chemical AMPK α inhibition, which is consistent with the AMPK α gene's inactivation. These findings imply that AMPK regulates adult worm glycogen stores, affecting both glycogen utilization and synthesis. This study suggests that AMPK should be further researched as a potential drug target, particularly for treatments aimed at preventing the spread of schistosome infection.

## Author contributions

All authors listed have made a substantial, direct, and intellectual contribution to the work and approved it for publication.

## Conflict of interest

The authors declare that the research was conducted in the absence of any commercial or financial relationships that could be construed as a potential conflict of interest.

## Publisher's note

All claims expressed in this article are solely those of the authors and do not necessarily represent those of their affiliated organizations, or those of the publisher, the editors and the reviewers. Any product that may be evaluated in this article, or claim that may be made by its manufacturer, is not guaranteed or endorsed by the publisher.

